# Classic and current concepts in adrenal steroidogenesis: a reappraisal

**DOI:** 10.20945/2359-3997000000438

**Published:** 2022-01-01

**Authors:** Claudio E. Kater, Rafael B. Giorgi, Flavia A. Costa-Barbosa

**Affiliations:** 1 Universidade Federal de São Paulo Escola Paulista de Medicina Departamento de Medicina São Paulo SP Brasil Unidade de Adrenal e Hipertensão; Laboratório de Esteroides, Divisão de Endocrinologia e Metabolismo, Departamento de Medicina, Escola Paulista de Medicina, Universidade Federal de São Paulo (EPM-Unifesp), São Paulo, SP, Brasil; 2 Universidade Federal de São Paulo Escola Paulista de Medicina Departamento de Medicina Brasil Divisão de Endocrinologia e Metabolismo, Departamento de Medicina, Escola Paulista de Medicina, Universidade Federal de São Paulo (EPM-Unifesp); Ambulatório de Adrenal, Divisão de Endocrinologia, Faculdade de Ciências Médicas e da Saúde, Pontifícia Universidade Católica de Sorocaba (PUC-Sorocaba), Sorocaba, SP, Brasil; Pontifícia Universidade Católica de Sorocaba Faculdade de Ciências Médicas e da Saúde Divisão de Endocrinologia Sorocaba SP Brasil; 3 Universidade Federal de São Paulo Escola Paulista de Medicina Departamento de Medicina São Paulo SP Brasil Divisão de Clínica Médica e Divisão de Endocrinologia e Metabolismo, Departamento de Medicina, Escola Paulista de Medicina, Universidade Federal de São Paulo (EPM-Unifesp), São Paulo, SP, Brasil

**Keywords:** Steroidogenesis, adrenal cortex, adrenocortical enzymes, adrenal disorders, glucocorticoids, mineralocorticoids, sex steroids, congenital adrenal hyperplasia, backdoor pathway, 11-oxigenated androgens

## Abstract

Adrenal steroid biosynthesis and its related pathology are constant evolving disciplines. In this paper, we review classic and current concepts of adrenal steroidogenesis, plus control mechanisms of steroid pathways, distribution of unique enzymes and cofactors, and major steroid families. We highlight the presence of a “mineralocorticoid (MC) pathway of zona fasciculata (ZF)”, where most circulating corticosterone and deoxycorticosterone (DOC) originate together with 18OHDOC, under ACTH control, a claim based on functional studies in normal subjects and in patients with 11β-, and 17α-hydroxylase deficiencies. We emphasize key differences between CYP11B1 (11β-hydroxylase) and CYP11B2 (aldosterone synthase) and the onset of a hybrid enzyme – CYP11B1/CYP11B2 –, responsible for aldosterone formation in *ZF* under ACTH control, in “type I familial hyperaldosteronism” (dexamethasone suppressible). In “apparent MC excess syndrome”, peripheral conversion of cortisol to cortisone is impaired by lack of 11β-hydroxysteroid dehydrogenase type 2, permitting free cortisol access to MC receptors resulting in severe hypertension. We discuss two novel conditions involving the synthesis of adrenal androgens: the “*backdoor pathway*”, through which dihydrotestosterone is formed directly from androsterone, being relevant for the fetoplacental setting and sexual differentiation of male fetuses, and the rediscovery of C19 11-oxygenated steroids (11-hydroxyandrostenedione and 11-ketotestosterone), active androgens and important markers of virilization in 21-hydroxylase deficiency and polycystic ovaries syndrome. Finally, we underline two enzyme cofactor deficiencies: cytochrome P450 oxidoreductase which partially affects 21- and 17α-hydroxylation, producing a combined clinical/hormonal picture and causing typical skeletal malformations (Antley-Bixler syndrome), and PAPSS2, coupled to SULT2A1, that promotes sulfation of DHEA to DHEAS, preventing active androgens to accumulate. Its deficiency results in reduced DHEAS and elevated DHEA and androgens with virilization. Future and necessary studies will shed light on remaining issues and questions on adrenal steroidogenesis.

## INTRODUCTION

Synthesis of steroid hormones by the adrenal cortices – as well as by the interstitial cells of the ovaries (theca cells) and testes (Leydig cells) –, is a complex and dynamic process whose mechanisms are still unclear. This is one possible reason why the study of steroidogenesis has always been involved by a mist of mystery and challenge for the non-specialist.

**Figure 1:**
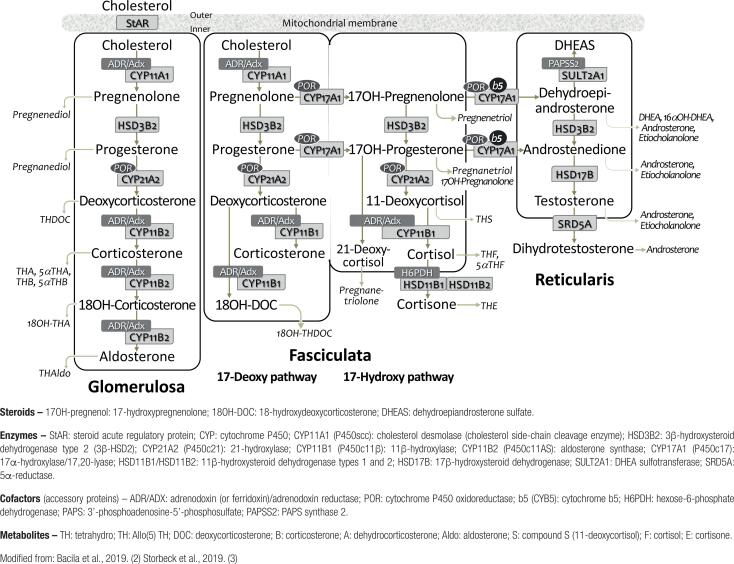
Current overview of adrenocortical steroid biosynthesis.

A contemporary concept of steroid biosynthesis as we understand it today is illustrated in Figure 1, which is somewhat different from the one(s) learned in classic textbooks on the subject. The graph illustrates (i) the most likely sequence of steroids formation, (ii) relevant intermediates in each pathway and respective layer of the adrenal cortex, (iii) specific enzymes and cofactors involved in the process and, (iv) the main urinary steroid metabolites.

In this paper, instead of presenting detailed physiologic and molecular mechanisms of steroidogenesis, we will review helpful concepts aiming at aspects that usually cause doubts and confusion. Nevertheless, for those interested in a more in-depth reading, we recommend consulting some excellent recent reviews (1-5).

## WHAT ARE STEROID HORMONES?

Steroid hormones constitute a large family of hormonal products – possibly more than 60 – that are synthesized largely by the adrenal cortices and the gonads, but also by the placenta and brain. Some are also metabolized or activated peripherally, mainly by the adipose tissue, the kidneys, the liver, and the skin (1,2,5).

Steroid hormones are classified according to their biochemical characteristics and relatively specific actions with names implying their major physiologic action: **glucocorticoids** (GC), **mineralocorticoids** (MC) and **sex steroids,** the latter with androgenic, estrogenic and progestogenic properties.

The basic molecular structure of all steroids (including vitamin D and bile acids) contains the cyclopentane-perhydrophenanthrene nucleus, which is also shared by their mutual precursor, cholesterol. Although derived from cholesterol, steroid hormones are not fat, but are found in the lipid fractions extracted from animals. They are lipo-soluble substances and, therefore, less water-soluble (1,2).

The term **pregnane** is assigned to cholesterol derivatives whose molecules contain 21 carbon atoms (C21); and **androstane** (C19) and **estrane** (C18) to those with 19 and 18 carbon atoms, respectively. The former gives rise to GC and MC, whereas androgens and estrogens derive from the other two (3,4).

The official nomenclature for steroids describes the chemical names of these compounds, which are extensive and complex (e.g., cortisol is: 11β,17α,21‐trihydroxy-pregn‐4‐ene‐3,20‐dione or 4-pregnene-11β,17α,21-triol-3,20-dione). Herein, we will use only the standardized trivial name or abbreviation of the steroid. Table 1 shows the trivial and chemical names, and abbreviations of the major adrenal steroid hormones and their urinary metabolites. For ease, the **term corticoid** will be used throughout instead of the more appropriate corticosteroid or adrenocorticosteroid.

**Table 1:** Nomenclature of adrenocortical steroids and main urinary metabolites sorted by classes, with trivial and full chemical names, and common abbreviations. (modified from reference 3)

Serum steroids: Trivial name [Chemical name]	Abbreviation		Main urinary metabolites Trivial name (unconjugated form)	Abbreviation
**General precursors:**				
Pregnenolone [5-pregnene-3β-ol-20-one]	**PREG; P5**		5-Pregnenediol	**5PD**
Progesterone [4-pregnene-3,20-dione]	**PROG; P4**		Pregnanediol	**PD**
17α-hydroxypregnenolone [5-pregnene-3β,17α-diol-20-one]	**17Preg; 17OHPreg; 17P5**		5-Pregnenetriol	**5PT**
17α-hydroxyprogesterone [4-pregnene-17α-ol-3,20-dione]	**17OHP; 17OHProg; 17P4**		Pregnanetriol	**PT**
**Glucocorticoids and their precursors:**				
11-Deoxycortisol [4-pregnene-17α,21-diol-3,20-dione]	**S**		Tetrahydro-11-deoxycortisol	**THS**
21-Deoxycortisol [4-pregnene-11β,17α-diol-3,20-dione]	**21dF**		Pregnanetriolone	**PTONA**
Cortisol [4-pregnene-11β,17α,21-triol-3,20-dione]	**F**		6β-Hydroxycortisol	**6β-OHF**
			Cortisol	**F**
			Tetrahydrocortisol	**THF**
			5α-Tetrahydrocortisol	**5α-THF**
			α-Cortol; β-Cortol	
			11β-Hydroxyeticholanolone	**11β-OHEt**
Cortisone [4-pregnene-17α,21-diol-3,11,20-trione]	**E**		Cortisone	**E**
			Tetrahydrocortisone	**THE**
			5α-Cortolone; β-Cortolone	
			11-Ketoetiocholanolone	**11ketoEt**
**Mineralocorticoids and their precursors:**				
11-Deoxycorticosterone [4-pregnene-21-ol-3,20-dione]	**DOC**		Tetrahydro-11-deoxycorticosterone	**THDOC**
Corticosterone [4-pregnene-11β,21-diol-3,20-dione]	**CORT; B**		Tetrahydro-11-dehydrocorticosterone	**THA**
			5α-Tetrahydro-11-dehydrocorticosterone	**5α-THA**
			5β-Tetrahydro-corticosterone	**THB**
			5α-Tetrahydro-corticosterone	**5α-THB**
18-Hydroxycorticosterone [4-pregnene-11β,18,21-triol-3,20-dione]	**18OHCORT; 18OHB; 18B**		18-Hydroxytetrahydro-11-dehydrocorticosterone	**18OHTHA**
Aldosterone [4-pregnene-11β,21-diol-3,20-dione-18-al]	**ALDO**		Tetrahydroaldosterone	**THAldo**
**Androgens and their precursors and metabolites:**				
17-Hydroxyallopregnanolone [5α-pregnane-3α,17α-diol-20-one]	**5α-17HP**		17-Hydroxyallopregnanolone	**5α-17HP]**
Dehydroepiandrosterone sulfate [5-androstene-3β-sulfate-17-one]	**SDHEA**	}	Dehydroepiandrosterone	**DHEA]**
Dehydroepiandrosterone [5-androstene-3β-ol-17-one]	**DHEA**		16α-Hydroxydehydroepiandrosterone	**16α-OHDHEA**
Androstenedione [4-androstene-3,17-dione]	**A4**	}	Androsterone	**An; AST**
Testosterone [4-androstene-17β-ol-3-one]	**T**		Etiocholanolone	**Et**
11β-Hydroxyandrostenedione [4-androstene-11β-ol-3,17-dione]	**11OHA4; 11β-OHA4**		11β-Hydroxyandrosterone	**11β-OHAn; 11βOHAST**
11β-Hydroxytestosterone [4-androstene-11β,17β-diol-3-one]	**11OHT**		11β-Hydroxyandrosterone	**11β-OHAn; 11βOHAST**
5α-Dihydrotestosterone [5α-androstane-17β-ol-3-one]	**DHT; 5α-DHT**		Androsterone	**An; AST**
“Hybrid” steroids:				
18-Hydroxycortisol [4-pregnene-11β,17α,18,21-tetrol-3,20-dione]	**18OHF**		18-Hydroxycortisol	**18OHF**
18-oxo-Cortisol [4-pregnene-11β,17α,21-triol-3,20-dione-18-al]	**18oxoF**		18-oxo-Tetrahydrocortisol	**18oxoTHF**

## THE ADRENAL CORTEX

Three physiologically distinct groups of corticoids are produced selectively (via specific pathways) by histologically and functionally different cell zones of the adrenal cortex:

- **zona glomerulosa** (ZG) – the outermost layer, just below the adrenal capsule – is composed of small, rounded cells that resemble glomeruli; *ZG* produces MC, whose main product is aldosterone, responsible for sodium and fluid retention, and control of blood pressure and electrolyte balance;
- **zona fasciculata** (ZF) – the thicker intermediate layer – contains lipid-laden cells available in bundles; *ZF* produces GC – the final product being cortisol –, that acts in numerous metabolic, hemodynamic, and inflammatory processes; and,
- **zona reticularis** (*ZR*) – the innermost layer, in contact with the adrenal medulla - whose cells resemble a cellular network; *ZR* produces adrenal androgens (e.g., dehydroepiandrosterone [DHEA], DHEA sulfate, androstenedione, and 11-oxygenated androgens), which stimulate pubertal development, androgen-dependent hairiness and libido.

The relative contribution of these layers for the total mass of the adrenal cortex is 10%, 75% and 15%, respectively (1).

### Control and selectivity of steroid production

Regulation of adrenal steroid biosynthesis is both external – through trophic stimulus by specific secretagogues (e.g. ACTH, angiotensin), as well as by innervation from the autonomous nervous system – and internal – via the presence and catalytic activity of several specific steroidogenic enzymes distributed throughout the adrenocortical layers. This combination ensures proper stimulation and selectivity of steroid hormones production (1-5).

**ACTH.** Adrenocortical trophic hormone (ACTH) is produced and released by the anterior pituitary, following a circadian rhythm determined by a genetically operated timekeeping system, referred to as “biological clock”, originated in the suprachiasmatic nucleus of the anterior hypothalamus; in stressful situations, the hypothalamic-pituitary-adrenal axis is greatly activated (6). Upon release, ACTH binds and interacts with specific G protein-coupled receptors (MC2R) located in the outer membrane of the adrenocortical cells; its action is mediated by activation of cAMP which occurs predominantly at *ZF* and *ZR*, although *ZG* also responds sharply to its stimulation (7).

The onset of ACTH action determines uptake of LDL-cholesterol from circulation and/or its synthesis from acetate stored in intracellular lipid droplets (cholesterol ester stores). This action results in intense and rapid intracellular trafficking of steroid precursors between the cytosol and mitochondria and triggers a series of hydroxylation and oxy-reduction reactions, adding oxygen (as hydroxyl groups) to the steroid precursor, rendering it increasingly specific, and ending with formation of the biologically active products (1,2,7).

**Angiotensin II.** Angiotensin II is the end-product of activation of the renin-angiotensin system (RAS); along with serum sodium and especially potassium concentrations, are the specific trophic stimuli of *ZG* (7,8). Action begins with its binding to specific *ZG* cell membrane receptors (AT2R) and activation of biosynthesis. Aldosterone is synthesized through specific enzymes of *ZG* leading to fluid and electrolyte regulation at the renal collecting tubules, epithelial cells of the gastrointestinal tract and salivary and sweat glands.

### Steroidogenic enzymes

Steroid synthesis begins with the acceleration of cholesterol flow through the mitochondrial membrane mediated by StAR (acute steroidogenic regulatory protein) (5,9). Cleavage of its side chain occurs within the mitochondria by CYP11A1 (P450scc, which acts as a lyase or desmolase) resulting in pregnenolone.

Typically, this is when the string of events takes place leading eventually to formation of biologically active steroids; in brief, pregnenolone moves to the smooth endoplasmic reticulum (SER) to be transformed in progesterone by action of 3β-hydroxysteroid dehydrogenase type 2 (HSD3B2). Then, a series of reactions catalyzed by monooxygenases (hydroxylases specific to their cortical layers) promote additional changes in the molecule, yielding several specific end-products.

Steroidogenic enzymes belong to two major groups: cytochromes P450 (CYP) and hydroxysteroid-dehydrogenases (ketosteroid-reductases) (HSD/KSR), both playing key roles in steroid biosynthesis (9,10).

CYP typically contains a prosthetic “heme” group (Fe^++/^Fe^+++^) that permits activation of molecular oxygen necessary to catalyze oxidative reactions, which are irreversible since CYP are functionally one-way (5,9). CYP are subdivided into: type 1 enzymes, located in the mitochondria, and type 2, in the SER. Each type is associated with different cofactors: CYP type 1, with ferredoxin or adrenodoxin (FDX1/ADX) and ferredoxin-reductase (FDXR/ADXR), and CYP type 2, with P450-oxidoreductase (POR) (Figure 1).

Six CYP participate in steroidogenesis: the first 3 are type 1, and the others are type 2 enzymes: 1) **CYP11A1** (or P450scc – cholesterol side-chain cleavage); 2) **CYP11B1** (or P450c11 – 11β-hydroxylase); 3) **CYP11B2** (or P450c11AS – aldosterone synthase); 4) **CYP17A1** (or P450c17 – 17α-hydroxylase); 5) **CYP21A2** (or P450c21 – 21-hydroxylase); and 6) **CYP19A1** (or P450aro – aromatase). CYP enzymes metabolize more than one substrate, thus being involved in multiple steps of steroid synthesis. This fact illustrates the broad and varied pathophysiologic scenario of the enzymatic defects of steroidogenesis (9,10).

HSD/KSR enzymes reversibly catalyze their reactions: although *in vitro* they work in both directions, *in vivo* they direct the steroid flow preferably to oxidative or reductive mode (9). HSD/KSR require NADH/NAD^+^ or NADPH/NADP^+^ as cofactors for their reductive and oxidative reactions, respectively.

HSD are classified into 2 classes based on their activity: dehydrogenases, that use NAD^+^ to oxidize hydroxysteroids to ketosteroids, and reductases, that use NADPH to reduce ketosteroids to hydroxysteroids. HSD involved in steroid synthesis that are relevant for human pathology include: 1) **HSD3B2** (or 3β-hydroxysteroid dehydrogenase type 2); 2) **HSD11B1** and **HSD11B2** (or 11β-hydroxysteroid dehydrogenase type 1 and type 2); and 3) **17-HSD** (several 17-hydroxysteroid dehydrogenases) (2,9,10).

### Pathways of adrenal steroidogenesis (Figure 1)

The first step of steroidogenesis takes place inside the mitochondria via conversion of cholesterol into pregnenolone, the common precursor of steroid hormones. This process involves 3 sequential monooxygenase reactions, all catalyzed by CYP11A1 (P450scc): two hydroxylation steps – at carbons 20 and 22 –, forming 20α,22-dihydroxy-cholesterol, followed by the cleavage of the C20-C22 bond, to form pregnenolone (3,9).

**Synthesis of mineralocorticoids**. In *ZG*, pregnenolone is irreversibly converted to progesterone by HSD3B2 which is then converted into 11-deoxycorticosterone (DOC) by CYP21A2. At this point, DOC serves as a substrate for CYP11B2 (aldosterone synthase), an enzyme that has activities of 11-hydroxylase, 18-hydroxylase, and 18-oxidase, thus catalyzing the 3 final steps that result in aldosterone formation (8,9).

Adrenal *ZG* provides a favorable enzymatic *milieu* for the synthesis of MC, as it is the only layer that expresses CYP11B2, and where CYP17A1 (the enzyme that directs pregnenolone towards the synthesis of GC and androgens), is virtually absent (8-10). Thus, this synthetic pathway produces 17-deoxy-steroids, in contrast to 17-hydroxy-steroids of the glucocorticoid pathway, typical of *ZF*.

Of interest, a similar 17-deoxy-pathway also occurs in the *ZF* – designated “MC pathway of *ZF*” –, in which the same sequence of events occurs until formation of DOC. At this point, however, DOC now serves as substrate for CYP11B1 (with activity of 11-hydroxylase and 18-hydroxylase), being separately converted into corticosterone (B) and 18-hydroxy-deoxycorticosterone (18OH-DOC) (7,8). Later we will discuss this route in more detail.

**Synthesis of glucocorticoids**. Synthesis of GC (17-hydroxy-steroids) occurs in the adrenal *ZF,* starting with 17-hydroxylation of pregnenolone to form 17-hydroxypregnenolone, catalyzed by CYP17A1, which is also responsible for the conversion of progesterone into 17-hydroxyprogesterone (17OHP). HSD3B2 also converts 17-hydroxypregnenolone into 17OHP, which is transformed into 11-deoxycortisol (compound S) by CYP21A2. Finally, CYP11B1 completes the process converting 11-deoxycortisol into cortisol.

Cortisol (or hydrocortisone) is the most important steroid of adrenal physiology, having multiple actions aiming at maintenance of homeostasis. In addition, cortisol blocks the secretion of its controller – ACTH – in the hypothalamic-pituitary axis by *the negative feedback* mechanism (2,7).

**Synthesis of androgens**. Synthesis of sex steroids occurs primarily in the adrenal *ZR* and the gonads. Pregnenolone is converted to 17-hydroxypregnenolone which is then transformed in dehydroepiandrosterone (DHEA); both reactions are catalyzed by CYP17A1, that has activities of 17-hydroxylase and 17,20-lyase (2,8-10). This latter CYP17A1 activity is backed by a critical cofactor, cytochrome b5 (CYB5), an electron transporter hemoprotein expressed only in the *ZR*. (4,9). 17-hydroxyprogesterone (17OHP) is also converted by CYP17A1 to androstenedione, but less efficiently than the former reaction.

DHEA is as precursor of active androgens, being converted into androstenedione by HSD3B2. However, part of the DHEA is converted into DHEA sulfate (DHEAS), a reaction catalyzed by a sulfotransferase (SULT2A), which requires the presence of PAPSS2 (3’-phosphoadenosine-5’-phosphosulfate synthase type 2) as cofactor (2,3,9). Sulfation of DHEA into DHEAS regulates the amount of DHEA available for androgen synthesis (11,12). Besides its role as peripheral precursor of more potent androgens, DHEA and DHEAS can be synthesized in the brain, where they play specific actions as neuro-steroids (11).

Two enzymes of the 17-hydroxysteroid dehydrogenase family are involved in androgen synthesis: 17-hydroxysteroid dehydrogenase type 3 (HSD17B3), that catalyzes the conversion of androstenedione to testosterone in the testes, and 17-hydroxysteroid dehydrogenase type 5 or aldo/ketoreductase C3 (HSD17B5 or AKR1C3), responsible for the synthesis of testosterone in the adrenals (12,13). Testosterone released into circulation is peripherally activated to dihydrotestosterone (DHT) by 5α-reductase type 2 (SRD5A2) (9,10,12). Later we will examine the synthesis of 11-oxygenated androgens.

The onset of adrenal androgen is clinically manifested by occurrence of adrenarche, as part of the *ZR* development; this evolving process begins in childhood but becomes apparent only after 6-8 years of age (10,13). *ZR*is characterized by an increase in the expression of cytochrome b5 and SULT2A, as well as a reduction in the expression of HSD3B2, a combination that favors synthesis of DHEA and DHEAS (9,10,12).

Recently, an alternative route of androgen synthesis – known as the “*backdoor pathway*” – has been identified in humans, which enables the synthesis of DHT directly from androsterone, without going through testosterone (5,12). (see below)

### Inconsistency of the steroid biosynthesis process as per the classic view

Synthesis of cortisol involves 17-hydroxylation (CYP17A1) of progesterone in the mitochondria, followed by 21-hydroxylation (CYP21A1) in SER to form 11-deoxycortisol, and subsequent 11-hydroxylation (CYP11B1), also in the mitochondria, resulting in its final synthesis and release.

This intense and winding intracellular trafficking of steroid intermediates between mitochondria and SER is a lengthy and seemingly illogical process, once synthesis and release of final steroids into circulation (considering that adrenocortical cells do not store hormones) is necessarily a sharp and efficient process, since cortisol is essential to confront acute stress.

Because of this apparent paradox, Lieberman and cols. (14-16) proposedin the 1980's that all machinery for specific steroid synthesis be contained in a single intracellular unit, within which the entire process – from the initial precursor (pregnenolone) to the biologically active final products (cortisol, aldosterone, DHEA) – would be carried out in full.

In this heuristic proposal, specific cell clusters expected to produce distinct steroids would contain “*hormonads*”, highly efficient steroid-producing mini-factories using multiple combined reactions. The term was coined by contraction of the words “*hormone* + *monads*”, the latter a philosophical concept of simple, fundamental, indivisible unity or a singular metaphysical entity! (www.collinsdictionary.com/pt/dictionary/english/monad) (14-16).

### Concept of “mineralocorticoids from the zona fasciculata”

Several physiological and pathophysiological aspects related to the synthesis and biological behavior of adrenal steroids cannot be reasonably explained by the simplistic and merely didactic idea of steroidogenesis, as currently taught. Several authors disagree with this dated view, proposing more plausible alternatives (4,5,7,13,17,18).

The main adrenal steroid, cortisol, is a product unique to the *ZF* under ACTH control. The second GC of physiological importance in man, corticosterone (B) – the main GC of rodents – is secreted in appreciable amounts also by *ZF*, under ACTH control. Although B is a required intermediate of the aldosterone synthetic pathway in *ZG* under RAS regulation, almost all its production and circulating levels originate from *ZF* and are essentially established by ACTH (7,18).

Likewise, the second MC of physiological importance after aldosterone is DOC which, although also a mandatory intermediate of the synthetic pathway of aldosterone in *ZG*, is virtually all derived from *ZF*, with circulating levels essentially determined by ACTH.

The following physiological evidence strengthen this assumption: (i) DOC secretion is amplified by exogenous ACTH stimulation and easily blocked by dexamethasone administration and, (ii) maneuvers aimed towards the RAS do not alter its production: volume expansion by saline infusion, high-sodium diet and fludrocortisone do not reduce DOC levels, whereas postural stimulation, diuretics, low-sodium diet, and angiotensin III infusion do not substantially increase its concentration (3,7,8,17,18).

Studies of the steroid profile of patients with congenital adrenal hyperplasia due to deficiencies of 17α-, 11β- or 21-hydroxylase (and POR), corroborate this physiological data, reinforcing the concept of a “MC (or non-17-hydroxylated) pathway of *ZF*” (3,7-9,18). Thus, although commonly referred to as a product of *ZG*, DOC must be regarded, for coherence and compelling respect to physiology, a “mineralocorticoid of *ZF*” (7,8,18).

Thus, as B and DOC are both of *ZF* origin, having circulating levels established by ACTH, independent of the RAS, it is senseless to maintain the “classic” view of steroidogenesis (in which B and DOC appear solely as intermediate products of the aldosterone pathway in the *ZG*). This concept not only confounds the non-specialist, but also leads to misinterpretation of adrenal steroid physiology.

It is worth mentioning that another exclusive product of the “MC pathway of *ZF*” – 18-hydroxy-DOC (18OHDOC) –, is synthesized from DOC by CYP11B1 (with activities of 11- and 18-hydroxylases), under ACTH control (7,8,18) (Figure 1). Thus, B, DOC and 18OHDOC – products of the non-17-hydroxy pathway of *ZF* – behave physiologically in parallel with cortisol and ACTH: they present circadian rhythms, are stimulated by ACTH, suppressed by dexamethasone, and are not influenced by RAS changes.

### CYP11B1 and CYP11B2: Similarities and differences

CYP11B1 (P450c11; 11β-hydroxylase) and CYP11B2 (P450c11AS; aldosterone synthase) are monooxygenases expressed respectively in the *ZF* (and *ZR*) and *ZG*, where they perform similar activities of 11β- and 18-hydroxylation. CYP11B1 catalyzes the final synthesis of cortisol and corticosterone from 11-deoxycortisol (S) and DOC, respectively, whereas CYP11B2 is typically responsible for production of aldosterone from DOC (Figure 1). Both localize in the inner mitochondrial membrane; both are encoded by contiguous genes, *CYP11B1* and *CYP11B2,* located on chromosome 8 (8q24.3), and have a high degree of homology (>94%). The similarities however end here (1,2,9,19).

Maybe for the similarity in their designation and because they exert similar catalytic actions on identical substrates, these enzymes are commonly (but incorrectly) believed as one. However, for reasons not totally understood (but likely due to formation of different intermediates), CYP11B1 has no 18-oxidase activity, being unable to form 18-hydroxycorticosterone (18OHB) under physiologic conditions. In only one condition – 17α-hydroxylase deficiency (17OHD) –,when the entire non-17-hydroxypathway of *ZF* (and only it) is significantly stimulated, 18OHB can be produced in substantial quantities since the *ZG* synthetic pathway is turned off and aldosterone suppressed (7,8,18).

*ZG* CYP11B2 catalyzes 3 sequential steps from DOC, generating B, 18OHB and, finally, aldosterone. It is remarkable that only CYP11B1 can hydroxylate DOC at C-18 position to form 18OHDOC (8,18).

In “type 1 familial hyperaldosteronism” (FH-1, or “dexamethasone suppressible”) a peculiar fact supports this knowledge. During meiosis, an unequal crossing-over between *CYP11B1* and *CYP11B2* genes occurs upon pairing of the two chromosome 8 haplotypes, favoring formation of a hybrid gene – *CYP11B1/CYP11B2* –, consisting of the *CYP11B1* promoter region and *CYP11B2* coding region (19,20). Hence, a new chimeric enzyme is encoded, with functional properties identical to that of CYP11B2 (able of synthesize aldosterone) but expressed in *ZF* under ACTH control. In this condition, excess aldosterone production results in a state of “primary” (RAS independent) hyperaldosteronism, that can be attenuated by treatment with dexamethasone or other GC. This disease usually manifests at an early age (<20y), with moderate to severe hypertension and risk of fatal cerebrovascular hemorrhages from aneurysm rupture (19,20).

### Conditions in which B and DOC production are relevant

The clinical importance of B and DOC are revealed in 2 relatively unusual conditions – deficiencies of 17α- and 11bβ-hydroxylase (the only two hypertensive forms of CAH). In both, cortisol formation is impaired by the respective enzyme restriction, whereas production of aldosterone is abolished by an unstimulated *ZG* synthetic pathway (as a result from RAS suppression). Resultant elevation of ACTH substantially stimulates the “MC pathway of *ZF*” producing increased amounts of DOC which provides substrate for increased 18OHDOC production (7,8,18-20).

DOC excess promotes sodium and fluid retention and consequent MC-dependent hypertension. RAS suppression is associated with inactivation and atrophy of the *ZG* synthetic pathway, abolishing aldosterone formation.

Furthermore, in 17OHD serum levels of B reach values 100 times higher than normal, in the range of 15 to 50 mcg/dL (normal: 0.1 to 0.6 mcg/dL). Although its GC activity is 10 times lower than cortisol, its abundant concentration provides enough GC activity to prevent “addisonian” manifestations in the affected patient (3,7,8,20). Another major action of B in this condition is assuming control of the negative feedback upon pituitary ACTH, keeping its levels normal to slightly elevated in absence of cortisol (21).

### Cortisol as mineralocorticoid

Albeit not an abnormality of steroid biosynthesis, a peculiar aspect of peripheral steroid metabolism deserves consideration. Cortisol *per se* has intrinsic MC properties given its ability to bind and activate the MC receptor analogous to aldosterone. In this respect, an interesting physiological event continuously nullifies this action: the extra-adrenal enzyme 11β-hydroxysteroid dehydrogenase type 2 (HSD11B2; called “*guardian of the gate*”), protects the MC receptor from cortisol activation, converting it to cortisone, which is not recognized by the receptor. This characterizes a unique and previously unknown physiological feature: “receptor selectivity determined by a protective enzyme” (20,22).

Congenital deficiency of HSD11B2 (known as “apparent MC excess syndrome”) or induced by use of inhibitors of its activity (glycyrrhizic/glycyrrhetinic acid, from licorice extract), impairs cortisol metabolism to cortisone allowing its free access to the MC receptor, producing severe hypertension (since serum cortisol concentration is near 1,000 times higher than that of aldosterone) (20,22).

### Alternative pathways of androgens synthesis (the backdoor pathway)

Studies in marsupials (Tammar wallaby) – whose sexual differentiation occurs after birth and inside the maternal abdominal pouch – demonstrated that DHT can be formed directly from 17-hydroxypregnenolone, without necessarily using DHEA, androstenedione or testosterone as intermediates (4,12,23).

**Figure 2:**
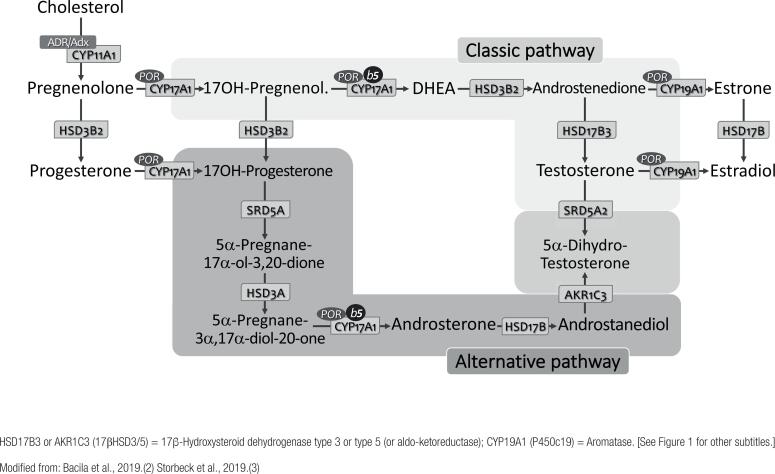
Production of adrenal androgens through the “classic pathway” and the “*backdoor pathway*” of biosynthesis.

This alternate fetoplacental pathway for androgen synthesis, called “*backdoor pathway*”, begins with formation of 17-hydroxyprogesterone (17OHP) that undergoes two reductions (at positions 5- and 3-) forming an intermediate compound (5α-pregnane-3α,17α-diol-20-one), followed by cleavage of the C17-C20 bond (catalyzed by the 17,20-lyase activity of CYP17A1 and CYB5), resulting in androsterone, which is finally converted to DHT. In contrast, the conventional pathway requires removal of the side chain from 17-hydroxypregnenolone (by CYP17A1 and CYB5), followed by 3β-oxidation, reduction at 17-keto by 17βHSD (HSD17B) and, finally, 5α-reduction (SRD5A) yielding DHT (Figure 2). This alternate path is active in the prenatal period of normal individuals and may play a role in male development during fetal life (12,23).

Recent evidence demonstrate involvement of the “*backdoor pathway*” in 21OHD, consequent to 17OHP excess. This pathway likely contribute to androgen excess leading to virilization of the female external genitalia in 21OHD; interestingly, activity of this androgen pathway appears to lessen after the first years of life. The “*backdoor pathway*” also contributes to hyperandrogenism observed in POR deficiency explaining, at least in part, maternal virilization during pregnancy in presence of placental aromatase and virilization of female fetuses, despite postnatal deficiency of sex steroids (12,23,24).

### The resurrection of adrenal C-19 11-oxygenated steroids (11-oxyandrogens)

**Figure 3:**
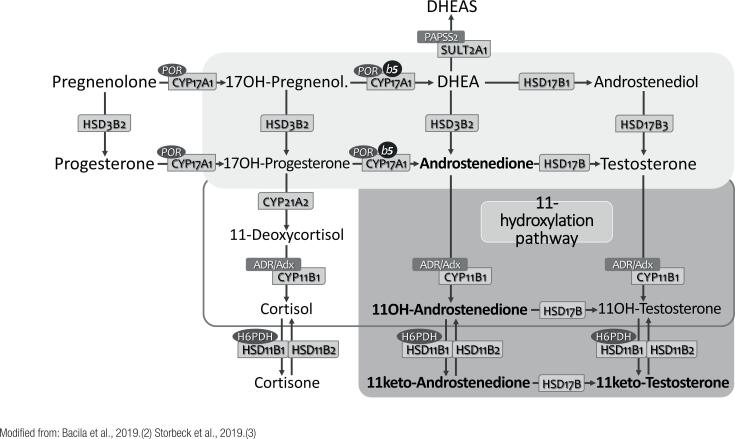
Synthesis of C19 11-oxygenated steroids (11-oxyandrogens) in the adrenal zona reticularis. [See Figure 1 for subtitles.]

C19 11-oxygenated steroids are a class of active adrenal androgens recognized 60+ years ago, which have been restudied recently. Both androstenedione and testosterone undergo 11-hydroxylation in the *ZR*, catalyzed by CYP11B1 (which is expressed in both *ZR* and *ZF*), resulting in 11-hydroxyandrostenedione (11-OHA4) and 11-hydroxytestosterone (11-OHT), respectively. Both steroids are subsequently reduced by HSD11B2 to 11-ketoandrostenedione (11-KA4) and 11-ketotestosterone (11-KT), respectively; 11-KT can still be 5α-reduced to form 11-keto-dihydrotestosterone (11-KDHT) (24-26) (Figure 3).

Although identified decades ago, 11-OHA4 has received minor attention, since it was believed that it would be part of an inactivation route of adrenal androgens. However, new evidence identified the C19 11-oxigenated steroids as a distinctive and relevant group of adrenal-derived androgens.

Different from 11-OHA4 – a peripheral precursor of the more active androgens –, both 11-KT and 11-KDHT are potent androgens, binding and activating the androgen receptor (AR) similarly to DHT. In addition, 11-KT seems to have a relevant physiological role during adrenarche, being the most important circulating androgen in females, followed by testosterone and 11-OHT. Recent studies reinforce the role of C19 11-oxygenated steroids in conditions of adrenal androgen excess. High concentrations of 11-OHT, 11-KT, 11-OHA4 and 11-KA4 were observed in patients with CAH due to 21OHD, suggesting that they have a major role in the clinical picture of hyperandrogenism of affected children (24-27).

Currently, the C19 11-oxygenated steroids are likely better markers of adrenal androgen excess in CAH than DHEA, androstenedione and testosterone, classically employed to monitor therapeutic control. C19 11-oxygenated steroids are also increased in polycystic ovary syndrome (PCOS), being correlated with markers of metabolic risk, such BMI and HOMA-IR index (24-27).

Thus, the potential clinical utility of this steroid group can be anticipated as future biomarkers for monitoring androgen excess in CAH and PCOS.

### Co-factors deficiency affecting enzymatic reactions of steroidogenesis

Several steroidogenic enzymes use co-factors to accomplish their reactions, basically providing electrons for hydroxylation reactions; among them, ADR/ADX (or FDR/FDX), POR, PAPSS2, H6PDH and cytochrome b5. We will discuss the deficiency of two of these co-factors, that may affect the respective enzymatic reactions in which they are involved.

**POR**. Cytochrome P450 oxidoreductase (CYP_OR_ or POR) was lately identified as a participant in steroidogenesis, and POR deficiency – the 6^th^ and last form of CAH –, was subsequently characterized. It helped explain some apparent inconsistencies of patients with 21OHD presenting with special characteristics of 17OHD; hence, this condition was initially called “combined 21- and 17-hydroxylase deficiencies” (13,27). The phenotype is variable, with ambiguous genitalia (or DSD) in both sexes, large ovarian cysts, incomplete masculinization and delayed puberty in males coupled with maternal virilization during gestation of an affected fetus. Mild MC excess may cause hypertension that usually manifests at adolescence or early adulthood. As anticipated, the typical metabolome of POR deficiency shows a variegated pattern of deficiencies of 21- and 17-hydroxylases.

POR deficiency results from homozygous or compound heterozygous *POR* gene mutations, which codifies for an electron donor protein to various mitochondrial enzymes, especially CYP21A2 and CYP17A1, but also CYP19A1, CYP51A1, and CYP26A1-C1. The “founding” p.R457H mutation came from a Japanese community, but several other mutations have already been reported (28).

In addition to changes in sexual development in 46,XX and 46,XY newborns – that are accompanied by pubertal failure, adrenal dysfunction and maternal virilization during pregnancy –, POR deficiency also results in typical skeletal malformations, known as Antley-Bixler syndrome (27,28).

**PAPSS2**. The SULT2A1 enzyme (DHEA sulfotransferase) converts the androgen precursor DHEA into its sulfate ester DHEAS, which is inactive (Figure 1). This reaction prevents conversion of DHEA into active androgens escaping possible hyperandrogenism. Also, the catalytic action of SULT2A1 requires PAPS (3’-phosphoadenosine-5’-phosphosulfate) synthase 2 (PAPSS2), an efficient sulfate donor. Inactivating mutations in *PAPSS2* gene are associated with premature puberty, hyperandrogenism, and anovulation, with reduced DHEAS and elevated androgens levels. Thus, PAPSS2 deficiency is characterized as a monogenic cause of excess adrenal androgens (29,30).

### Final remarks

Adrenal steroidogenesis and its related pathology are ever developing disciplines, the progress of which is coupled with contemporary and innovative technology. We reviewed classic and current concepts of adrenal steroid biosynthesis, including intrinsic regulatory mechanisms and specific enzyme/co-factors required for production of distinctive steroid groups. We highlighted several concepts that have being reluctantly accepted, as mineralocorticoid production by zona fasciculata, which helps exlain the unique pathophysiology of CAH, particularly 17-hydroxylase deficiency, and recent information regarding production of adrenal androgens by alternative routes (the “*backdoor pathway*”), where dihydrotestosterone is formed from androsterone –, and the rediscovery of C19 11-oxygenated steroids, potent androgens and markers of virilization in CAH and PCOS. For years, the pathophysiology of rare adrenal diseases is being revealed, as “mother nature” provides continuous examples to endocrinologists and physiologists. Several open questions in this topic are being looked upon in the light of new molecular biology and genetic approaches. This review paper comes as an attempt to refresh former established concepts, introduce recent ones, and open new paths for researchers to unveil still unanswered questions.
